# Lithium enrichment from mine waters using CO_2_ hydrate-based desalination

**DOI:** 10.1038/s41598-026-43925-7

**Published:** 2026-03-17

**Authors:** Mahdieh Khajvand, Georgios Kolliopoulos

**Affiliations:** Department of Mining, Metallurgical, and Materials Engineering, Université Laval, 1065 Avenue de la Médecine, Québec City, QC G1V 0A6 Canada

**Keywords:** CO_2_ hydrate-based desalination, Lithium enrichment, Mine water treatment, Water recovery, Chemistry, Energy science and technology, Engineering, Environmental sciences

## Abstract

Lithium (Li) recovery from secondary resources, such as mine waters, is essential to meet the increasing global demand. However, lithium in mine waters typically occurs at low concentrations, necessitating efficient and environmentally sustainable methods for enrichment. Hydrate-based desalination (HBD) is a novel and energy-efficient approach that can simultaneously concentrate lithium and produce clean water. In this study, CO_2_ HBD was applied to upgrade lithium in real mine waters. The effects of stirring rate and reaction time were evaluated, showing that operation at 600 rpm for 1 h achieved a lithium enrichment factor of 1.57 ± 0.09 and a water recovery of 53 ± 4%. A multi-stage configuration further improved performance, with Li enrichment in the brine stream reaching 2.75. This increased the concentration of lithium from 180 mg/L to about 500 mg/L, a level considered feasible for further treatment towards lithium recovery. The desalting efficiency in the hydrate phase also increased from 40 to 81%. A key finding was that naturally occurring fine particles (silicate/aluminosilicate particles) in the mine water acted as effective *in situ* kinetic promoters, eliminating the need for external additives. This not only simplifies the process design but also helps to avoid additional separation steps and costs. Overall, these results highlight CO_2_ HBD as a promising technology for lithium enrichment from dilute aqueous resources and water reuse in mining operations, with potential to contribute to more sustainable resource management.

## Introduction

Mine water is generally considered an environmental and operational challenge in the mining industry^1^. Large volumes of water are used during extraction and processing, and once discharged, this water often carries high levels of dissolved salts, suspended solids, metals, organics, and other contaminants that can threaten surrounding ecosystems^1,2^. Consequently, effective treatment of mine water is essential to mitigate its environmental impact^3^. However, mine water is not only a challenge but also a potential resource. In addition to unwanted pollutants, it frequently contains dissolved metals that are typically lost with wastewater^4^. Among these, lithium has attracted particular attention due to its rising economic value and strategic importance for energy storage and battery technologies^5^. Recovering lithium from mine water therefore represents a dual opportunity. Reducing the environmental footprint of mining operations while simultaneously extracting a high-value element from what would otherwise be wasted.

The growing demand for lithium, driven primarily by its pivotal role in rechargeable batteries for electric vehicles and energy storage systems, has intensified interest in secondary resources such as mine water^6,7^. However, the lithium content of such waters is generally low, making a concentration step essential prior to purification and recovery^8^. A wide range of separation and concentration technologies has been investigated for this purpose. Proposed methods include electrodialysis^9–11^, reverse osmosis^12^, forward osmosis^13,14^, hydrate-based desalination^15,16^, and hybrid processes^17–19^. These processes act as concentration steps, raising lithium levels to a range that makes downstream recovery feasible. Each approach has specific advantages and limitations depending on factors such as energy demand, selectivity, scalability, and cost, highlighting the need for continued exploration of alternative technologies.

Membrane-based processes such as nanofiltration (NF), reverse osmosis (RO), forward osmosis (FO), and electrodialysis (ED) have been widely investigated for lithium recovery from saline and industrial waters^10–12,17–19^. Among these, RO is the most mature and widely applied at industrial scale. For example, Qiu et al. (2019) demonstrated that a hybrid RO–ED system can effectively recover lithium from produced water, where RO served as a crucial pre-concentration step to reduce energy consumption in the subsequent ED stage^17^. Although RO offers high rejection of salts, it requires high operating pressures (5–8.2 MPa) and is therefore energy-intensive^20^. FO has been proposed as a lower-energy alternative because it relies on an osmotic gradient rather than externally applied pressure^21^. However, the recovery of the draw solution remains energy-demanding, which limits the process’ overall efficiency^21,22^. ED and NF show promises for selective ion separation, yet they face similar challenges. A major limitation across all membrane-based technologies is fouling, which reduces performance and increases operational costs^23^.

While the above-mentioned methods provide valuable approaches for lithium separation and concentration, their inherent limitations highlight the need for alternative technologies. Hydrate-based desalination (HBD) is an emerging technique that exploits the formation of gas hydrates to separate fresh water from saline solutions such as mine waters^24^. Under suitable pressure and temperature conditions, gases like CO_2_ form crystalline clathrate hydrates in which water molecules organize into cage-like lattices that encapsulate gas molecules, while excluding dissolved salts and impurities. Once separated from the brine, these hydrates can be dissociated to release purified water. Among various hydrate-forming gases, CO_2_ offers several advantages for desalination applications. It forms hydrates under relatively mild operating conditions, can be sustainably sourced from industrial emissions, and provides stable hydrate structures that are easily separated from the produced water^25–27^. The brine stream generated during HBD contains concentrated salts and elements, including lithium, which are becoming more economically valuable. Thus, HBD not only enables desalination but also provides an opportunity for simultaneous resource recovery. Compared with conventional approaches, HBD consumes less energy and does not rely on membranes or adsorbents, making it a promising alternative for treating complex mine waters^20^.

Despite HBD’s advantages, practical applications for lithium concentration remain limited. To-date, only a few studies have explored lithium enrichment using HBD. Ling et al. (2020) investigated cyclopentane hydrate formation in synthetic brines using graphite powder to accelerate nucleation^15^. Although induction times were reduced, the need to remove the added graphite and the long reaction times (up to 14 h) remained major drawbacks^15^. A later study used chlorodifluoromethane (R22) as a hydrate former for lithium recovery and freshwater production, but R22’s high environmental impact and the need for strict gas recovery limit its practical use^28^. Another recent work examined CO_2_ hydrate formation in lithium salt solutions from a thermodynamic and structural perspective^29^. Overall, these studies show that HBD for lithium enrichment is still at an early stage, and additional research is required to improve process efficiency and practical feasibility.

In this context, a critical gap remains because most current studies rely on synthetic brines and external kinetic additives, which complicate post-treatment and industrial scaling. The present work demonstrates that naturally occurring suspended solids (e.g., cristobalite and albite) act as *in situ* kinetic promoters in a CO_2_ HBD process achieve both water purification and lithium enrichment from real mine waters. By leveraging the inherent mineralogy of the feed, this work provides a dual-objective solution for Li enrichment and water recovery that eliminates the need for synthetic promoters, offering a more sustainable and simplified process design for resource recovery in mining operations. These naturally present suspended solids were found to act as effective nucleation sites, significantly reducing the induction time and enabling successful hydrate formation within a 1 h experimental cycle. Building on this observation, the study further investigates the effects of stirring rate and reaction time on process efficiency, assesses a multi-stage operation for cumulative resource recovery, and examines the robustness of the process under varying initial lithium concentrations.

## Methodology

### Materials

High-purity carbon dioxide gas (99.995%, Praxair Canada Inc.) was used as the hydrate forming agent. The mine water used in this study, with its composition summarized in Table 1, was obtained from a mining facility located in Canada.

**Table 1:** Characteristics of the mine water used in this work.

Parameter	Value	Unit
General water quality		
pH	7.7 ± 0.1	–
Conductivity	6.84 ± 0.03	mS/cm
Total dissolved solids	3.35 ± 0.02	g/L
Total suspended solids	15 ± 3	mg/L
Cations		
Li	181 ± 4	mg/L
Na	937 ± 4	mg/L
K	145 ± 1	mg/L
Mg	14 ± 1	mg/L
Ca	67 ± 1	mg/L
Si	22 ± 1	mg/L
Anions		
Carbonated species	223 ± 12	mg/L
Cl	358 ± 18	mg/L
SO_4_	2592 ± 136	mg/L

### Experimental setup and general procedure

**Fig. 1:**
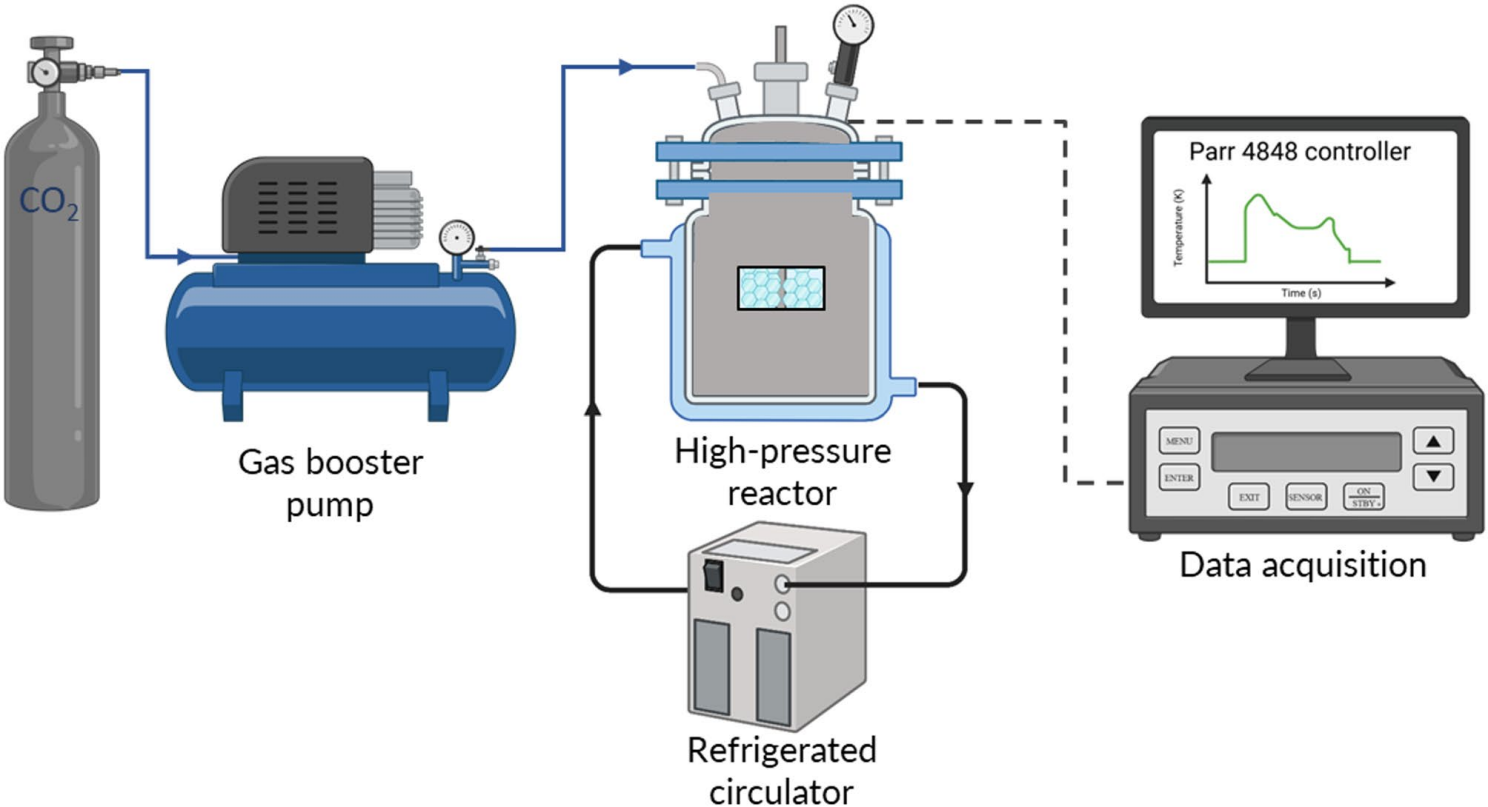
Schematic of the CO₂ hydrate-based desalination setup used in this work with high-pressure reactor, gas booster, refrigerated circulator, and data acquisition system.

A schematic of the experimental setup used in this study is shown in Fig. 1. It was designed to investigate gas hydrate formation at controlled temperature and pressure conditions using carbon dioxide as the guest gas. The system consists of a 1.8 L jacketed high-pressure stirred reactor constructed from Hastelloy C-276 (Parr Instrument Company, USA), a gas booster (Haskel International, Inc., USA) capable of delivering outlet pressures up to 62 MPa before entering the reactor, a refrigerated circulator (VWR International, LLC, USA) with a water–ethylene glycol mixture for temperature regulation, and a data acquisition unit for monitoring and controlling process variables (Parr 4848 controller). The reactor was equipped with pressure transducers and thermocouples for continuous monitoring, while gas–liquid mixing was achieved with an overhead mechanical stirrer operated at controlled speeds depending on the experimental design.

For each desalination experiment, 400 mL of mine water was introduced into the high-pressure reactor. Once the desired temperature was stabilized, the reactor was flushed with CO_2_ to remove residual air and then pressurized with CO_2_ to the operating conditions of 274.15 K and 3.58 MPa. These conditions were selected based on previous studies in this group^30,31^ and phase equilibrium predictions obtained using Multiflash software (version 7.4.17, KBC Advanced Technologies, A Yokogawa, UK, https://www.kbc.global/process-optimization/technology/simulation-software/multiflash-simulation-software/). During hydrate formation, CO_2_ consumption caused a gradual pressure decrease. Therefore, additional CO_2_ was supplied automatically through actuator valves to maintain constant pressure. At the end of each run, the reactor was depressurized, and the hydrate phase was separated from the residual brine by vacuum filtration. The collected hydrates were subsequently exposed to ambient conditions, allowing their dissociation to occur, and the hydrate melt was recovered. Both the hydrate-derived water and the brine were weighed and analyzed to evaluate water recovery, desalting efficiency, and Li enrichment.

### Experimental procedure

The hydrate-based desalination process was investigated through five sets of experiments, each designed to evaluate a specific parameter influencing hydrate formation kinetics and separation performance. All experiments followed the general procedure described in Sect.  2.2, with variations applied according to the objective of each assay.

The first set of experiments focused on assessing the role of suspended solids (SS) in hydrate nucleation. Mine water was tested at three filtration levels: (i) unfiltered (as-received), (ii) filtered through a 1.5 μm filter paper, and (iii) filtered through a 0.22 μm filter paper. In each case, 400 mL of feedwater was processed under identical operating conditions: 200 rpm stirring, 274.15 K, and 3.58 MPa for a duration of 1 h. This experimental design enabled direct comparison of induction time and hydrate formation behavior as a function of SS content.

The second set of experiments examined the influence of stirring rate on both kinetics and separation performance. Stirring rates of 200, 400, and 600 rpm were applied at otherwise constant conditions. Water recovery, desalting efficiency, and Li enrichment factor were determined to evaluate the interplay between enhanced kinetics and salt exclusion. The upper limit of 600 rpm was selected based on the mechanical constraints of the stirring system of our setup, which consisted of a pulley with a maximum operating speed of 600 rpm.

The third set of experiments evaluated how extending reaction time influences hydrate growth and separation. Assays were conducted at 600 rpm and standard operating conditions for 30, 60, and 90 min. In addition to the performance metrics, observations were made regarding hydrate rigidity and the ease of separating hydrates from brine at longer durations, as hydrate–brine separation became increasingly challenging after 90 min.

The fourth set of experiments investigated the performance of HBD in a multi-stage configuration. In the first stage, 400 mL of mine water was treated under identical operating conditions (temperature: 274.15 K, CO_2_ pressure: 3.58 MPa, stirring speed: 600 rpm, reaction time: 1 h). This treatment produced two product streams: hydrate and brine. In each subsequent stage, 400 mL of each stream were separately used as feedwater for new HBD experiments conducted under the same operating conditions. This multi-stage approach allowed the evaluation of cumulative water recovery, desalting efficiency, and Li enrichment, as well as a comparison of how the brine and hydrate streams evolved across a series of successive HBD stages.

The final series of experiments investigated the impact of lithium concentration in the feed solution. Mine water was spiked with LiCl to adjust the initial lithium concentration to 0.03 M (as-received, 180 mg/L), 0.10 M, 0.23 M, and 0.45 M. All runs were carried out under the optimal operating conditions identified from earlier experiments (600 rpm, 274.15 K, 3.58 MPa, 1 h). The results were analyzed to determine how lithium concentration influenced water recovery, desalting efficiency, and lithium enrichment factor.

### Performance parameters

Three key performance indicators were used to evaluate the HBD process including water recovery, desalting efficiency (in the hydrate phase), and Li enrichment (in the brine phase).

In each experiment, 400 mL of real mine water was used as the feed solution. After hydrate formation and separation steps, approximately 390 mL of liquid (hydrate melt + brine) was consistently recovered. This ~ 10 mL discrepancy was observed across all runs and is attributed to systematic handling losses, including liquid retention on the filter paper, reactor walls, and during transfer. To account for this, water recovery was calculated relative to the actual liquid recovered, according to Eq. (1):

1 $$Water\ recovery(\%) = \frac{V_{hydrate}}{V_{hydrate}+V_{brine}} \times 100$$

where, V_hydrate_ is the volume of water recovered from hydrate melting, and V_brine_ is the volume of residual brine. This definition eliminates the effect of systematic experimental loss and provides a reliable measure of hydrate separation efficiency.

Desalting efficiency was calculated based on the reduction in TDS concentration in the hydrate phase compared with the feed, using Eq. (2):

2 $$Desalting\ efficiency (\%) = \frac{C_{feed}^{TDS} - C_{hydrate}^{TDS}}{C_{feed}^{TDS}} \times 100$$

where ${\mathrm{C}}_{feed}^{TDS}$ and ${\mathrm{C}}_{hydrate}^{TDS}$ are the TDS concentrations in the feedwater and in the hydrate melt, respectively.

Li enrichment in the brine was evaluated as the ratio of lithium concentration in the brine to that in the feedwater, according to Eq. (3), where ${\mathrm{C}}_{brine}^{Li}$ is lithium concentration in the residual brine and ${\mathrm{C}}_{feed}^{Li}$ is initial lithium concentration in mine water.

3 $$Li\ enrichment\ factor = \frac{C_{brine}^{Li}}{C_{feed}^{Li}}$$

It is worth noting that the composition of the lost fraction (~ 10 mL) could not be determined (hydrate or brine). Since desalting efficiency and Li enrichment are concentration-based metrics, this volume loss was not included in these calculations. All experiments were repeated at least three times to ensure reproducibility, except for stage-3 of the multi-stage process. Stage-3 was performed only once because, although each stage requires 400 mL of feed, producing sufficient feed for stage-3 required multiple preceding experiments to accumulate adequate hydrate and brine, making repetition impractical.

### Analytical methods

The suspended solids were characterized using Fourier-transform infrared spectroscopy (FTIR), scanning electron microscopy (SEM), X-ray diffractometer (XRD), and X-ray fluorescence (XRF). FTIR spectra were obtained with a Cary 630 FTIR spectrometer (Agilent Technologies, USA) in attenuated total reflectance (ATR) mode, over the range 400–4000 cm^− 1^ with a resolution of ≤ 2 cm^− 1^. Surface morphology was examined with a VEGA3 SEM (Tescan, Czech Republic) operated at 10 kV. Elemental analysis was carried out using energy dispersive spectroscopy (EDS) equipped with an EDAX Element detector, operating across an X-ray energy detection of 0–10 keV with a resolution of 128.8 eV. The crystalline phases of the suspended solids were characterized using an Aeris X-ray diffractometer (PANalytical B.V., Almelo, the Netherlands). Measurements were performed with Cu Kα radiation (λ = 0.154 nm) operated at 40 kV and 8 mA. Diffraction patterns were collected over a 2θ range of 5° to 85°, with a step size of 0.02° and a counting time of 48 s per step. Phase identification was carried out using HighScore Plus software (version 4.9a, PANalytical B.V., Almelo, the Netherlands, https://www.malvernpanalytical.com/en/products/category/software/x-ray-diffraction-software/highscore-with-plus-option). The chemical composition of the SS was further analyzed by XRF using a Rigaku ZSX Primus II spectrometer (Rigaku Corporation, Japan). Particle size distribution and concentration of SS in the mine water were determined via nanoparticle tracking analysis (NTA), using a ZetaView BASIC NTA instrument (Particle Metrix, Germany). This technique is particularly suited for characterizing particles with sizes between 10 and 1000 nm and concentrations ranging from 10^6^ to 10^9^ particles/mL.

Dissolved cations were quantified using microwave plasma atomic emission spectroscopy (MP-AES, Agilent 4100 + upgraded to 4200 torch system, Agilent Technologies, USA) and inductively coupled plasma optical emission spectrometry (ICP-OES, Agilent 5110 Dual View, Agilent Technologies, USA). Dissolved anions were analyzed using a Thermo Scientific Integrion high-pressure ion chromatography (HPIC) system (Thermo Scientific, USA). Carbonated species were quantified using a Shimadzu VCPH total organic carbon (TOC) analyzer (Shimadzu Corporation, Japan). Basic water quality parameters, including pH, electrical conductivity, and total dissolved solids (TDS), were measured with a Thermo Scientific Orion Versa Star Pro multiparameter meter. TDS was estimated using the equivalent NaCl concentration. The determination of suspended solids followed the standard analytical protocol (MA 115 – S.S. 1.2). Samples were filtered through pre-conditioned and pre-weighed Whatman 934 AH filters. After filtration, the retained solids were oven-dried at 103–105 °C, then reweighed. The increase in filter mass, relative to its initial weight, was used to determine the amount of SS in the sample^32^.

## Results and discussion

### Suspended solids and their effect on the kinetics of the process

One of the main challenges of HBD is the inherently slow kinetics of gas hydrate formation. To overcome this limitation, various kinetic promoters have been explored including surfactants^33^ (e.g. sodium dodecyl sulfate and tetrahydrofuran), amino acids^34^ (e.g., phenylalanine, histidine, L-valine), bio-additives derived from plant materials^35^ (e.g. lotus, neem, or maize starch), nanoparticles^36^ (e.g. Al_2_O_3_, graphene oxide, and silica), cellulose^37^ and recently CO_2_ nanobubbles^25^. Kinetic promoters reduce the induction time and accelerate the hydrate growth rate by increasing the solubility of hydrate-forming gases in the liquid phase and lowering the gas–liquid interfacial tension, thereby facilitating faster nucleation and crystal growth^20^.

During the preliminary experiments, a notable variation in induction time was observed depending on the degree of mine water filtration. Specifically, the as-received mine water exhibited significantly shorter induction times compared to its filtered counterparts. This behavior can be explained by nucleation theory. Hydrate nucleation may proceed via homogeneous or heterogeneous pathways. Homogeneous nucleation occurs spontaneously within the bulk liquid and requires overcoming a relatively high interfacial energy barrier. In contrast, heterogeneous nucleation takes place at solid–liquid interfaces, such as suspended particles or reactor walls, where the presence of a third phase lowers the energy barrier for nucleus formation. Consequently, nucleation occurs more readily on these surfaces, leading to shorter induction times^38^. The suspended solids present in the mine water likely acted as effective heterogeneous nucleation sites, thereby enhancing hydrate formation kinetics.

**Fig. 2:**
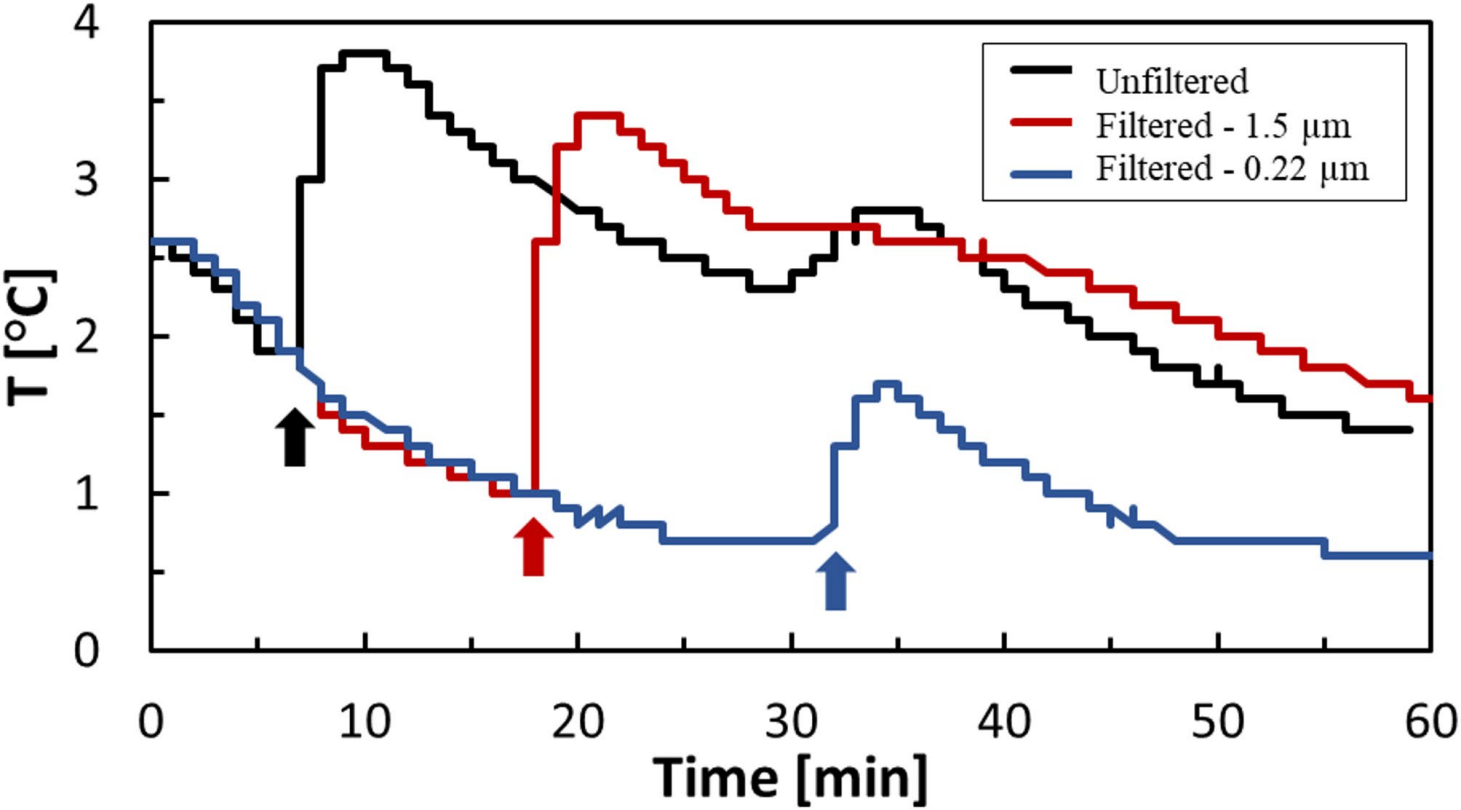
Temperature profile for CO_2_ hydrate formation in the mine water with and without filtration at 274.15 K, 3.58 MPa, and 200 rpm. ‘Unfiltered’ refers to the raw mine water. Filtration was performed using 1.5 μm and 0.22 μm filters to remove suspended solids.

To examine the effect of SS, HBD experiments were performed using mine water at three filtration levels: unfiltered (as-received), filtered through a 1.5 μm filter paper, and filtered through a 0.22 μm filter paper. The results revealed that the shortest induction time (7 min) occurred in the unfiltered sample, followed by the 1.5 μm filtered sample (18 min) and the longest induction time observed in the 0.22 μm filtered water (33 min). These findings suggest that the presence and size distribution of suspended particles plays a key role in hydrate nucleation, likely by facilitating heterogeneous nucleation. Figure 2 illustrates the temperature profiles for CO_2_ hydrate formation in mine water with and without filtration. The induction time was determined by observing a sudden increase in temperature, as hydrate formation is an exothermic process^25^.

The amount of SS in the mine water was measured to be approximately 15 mg/L, a low but potentially catalytically significant level. To investigate their composition and mineralogy, two methods were employed: filtration and centrifugation. In the former, mine water was filtered through a Whatman 934 AH filter according to the analytical procedure (MA 115 - S.S. 1.2) for the measurement of SS^32^. Both the clean and loaded filters were analyzed via XRF and SEM–EDS, to distinguish SS signal from filter background. In the second, mine water was centrifuged, and the resulting solids were dried in oven at 105 °C to evaporate the remaining water, which were then analyzed by SEM–EDS, XRD and FTIR. This method captured both suspended particles and salts crystallized from TDS during drying.

Table 2 presents the XRF results for the filtration method. The XRF analysis of the clean filter showed a high content of SiO_2_ (49%), which is consistent with the composition of the filter itself (as Whatman^®^ 934-AH discs are made of borosilicate glass microfibers). Other notable oxides detected in the clean filter include Al_2_O_3_ (13.8%), CaO (21.8%), B_2_O_3_ (6.3%), and MgO (2.5%). In the loaded filter, the same oxides (SiO_2_, Al_2_O_3_, CaO, and B_2_O_3_) were still present but in lower proportions. This decrease suggests that the suspended solids collected on the filter diluted the XRF signal from the underlying borosilicate-based matrix. The dominant additional component detected in the loaded filter was CO_2_ (32.6%), which likely represents carbonate minerals. Other components introduced by the suspended matter include Na_2_O (3.3%), SO_3_ (1.5%), K_2_O (1.4%), and nitrogen-containing compounds (2.0%), supporting the presence of both mineral and organic particulate matter in the mine water.

**Table 2:** XRF analysis of clean and loaded filters after mine water filtration (oxide form).

Component	Clean filter	Loaded filter
	Weight [%]	Weight [%]
B_2_O_3_	6.3	3.5
*CO_2_	2.8	32.6
N	–	2.0
Na_2_O	–	3.3
MgO	2.5	–
Al_2_O_3_	13.8	10.5
SiO_2_	49.1	37.0
**SO_3_	–	1.5
K_2_O	–	1.4
CaO	21.8	7.5
Remaining	3.7	0.7

*CO_2_ indicates the presence of carbonates.

**SO_3_ indicates sulfur oxide compounds, likely from sulfate species.

To better understand the composition of SS, SEM-EDS results for the clean filter, loaded filter, and centrifuged sample are summarized in Table 3. The clean filter, made of borosilicate glass microfiber, showed dominant peaks of Si (20.6%), O (47.3%), and Al (7.2%), which align with its composition. When analyzing the loaded filter, an increase in carbon (from 4.9% to 19.9%), the appearance of elements, such as Na (3.9%) and K (1.5%), and a decrease in Ca (from 16% to 2.8%), suggest deposition of organic matter and mineral particulates from the mine water.

The centrifuged sample, which captures both suspended particles and precipitated salts, showed significant amounts of Na (14.4%), Si (15.9%), Al (4.4%), and new signals for S (8.7%) and Cl (4.4%). These results indicate that Si and Al are present in both filtration and centrifugation methods, suggesting they exist in the mine water either as suspended particles or precipitated phases after drying. Although the exact form (dissolved vs. particulate) cannot be fully distinguished, their consistent detection across both methods reinforces their presence.

**Table 3:** SEM-EDS elemental composition of the clean filter, loaded filter after mine water filtration, and centrifuged mine water sample.

Element	Clean filter	Loaded filter	Centrifuged sample
	Weight [%]	Weight [%]	Weight [%]
C	4.9	19.9	–
O	47.3	45.8	43.9
Na	–	3.9	14.4
Mg	2.1	–	–
Al	7.2	6.3	4.4
Si	20.6	17.6	15.9
S	–	–	8.7
Cl	–	–	4.4
K	–	1.5	3.7
Ca	16.0	2.8	2.9
Remaining	1.8	2.3	1.6

**Fig. 3:**
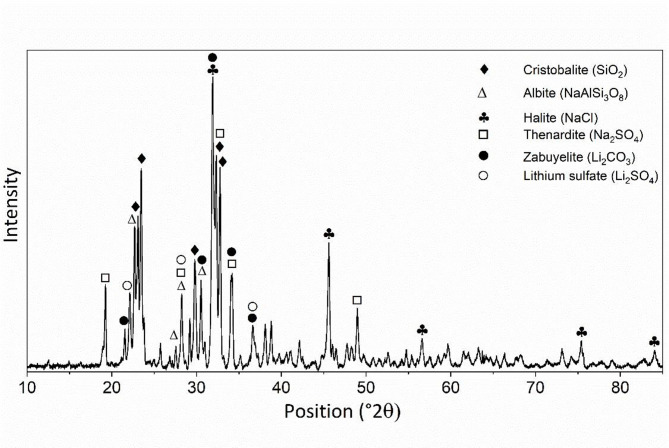
XRD pattern of suspended solids obtained from mine water centrifugation.

To identify the crystalline phases within the SS and address the potential for complex mineralogy, XRD analysis was performed on the centrifuged and dried mine water samples (Fig. 3; Table 4).

The XRD analysis identified the presence of cristobalite (SiO_2_) and albite (NaAlSi_3_O_8_). This confirms that the Al and Si detected by XRF and SEM–EDS originate from naturally occurring silicate and aluminosilicate minerals, rather than from discrete synthetic-type SiO_2_ or Al_2_O_3_ nanoparticles. In addition, the diffraction peaks corresponding to halite (NaCl), thenardite (Na_2_SO_4_), zabuyelite (Li_2_CO_3_) and lithium sulfate (Li_2_SO_4_) were observed. These phases are attributed to the crystallization of dissolved salts during sample drying at 105 °C prior to analysis.

**Table 4:** Identification of crystalline phases in mine water via XRD.

Reference code	Assigned mineral phase	Associated elements (from XRF/EDS/ICP)
96-901-6226	Cristobalite	Si, O
96-901-6733	Albite	Na, Al, Si, O
96-100-0042	Halite	Na, Cl
96-101-1185	Thenardite	Na, S, O
96-900-9643	Zabuyelite	Li, C, O
96-101-0468	Lithium sulfate	Li, S, O

**Fig. 4:**
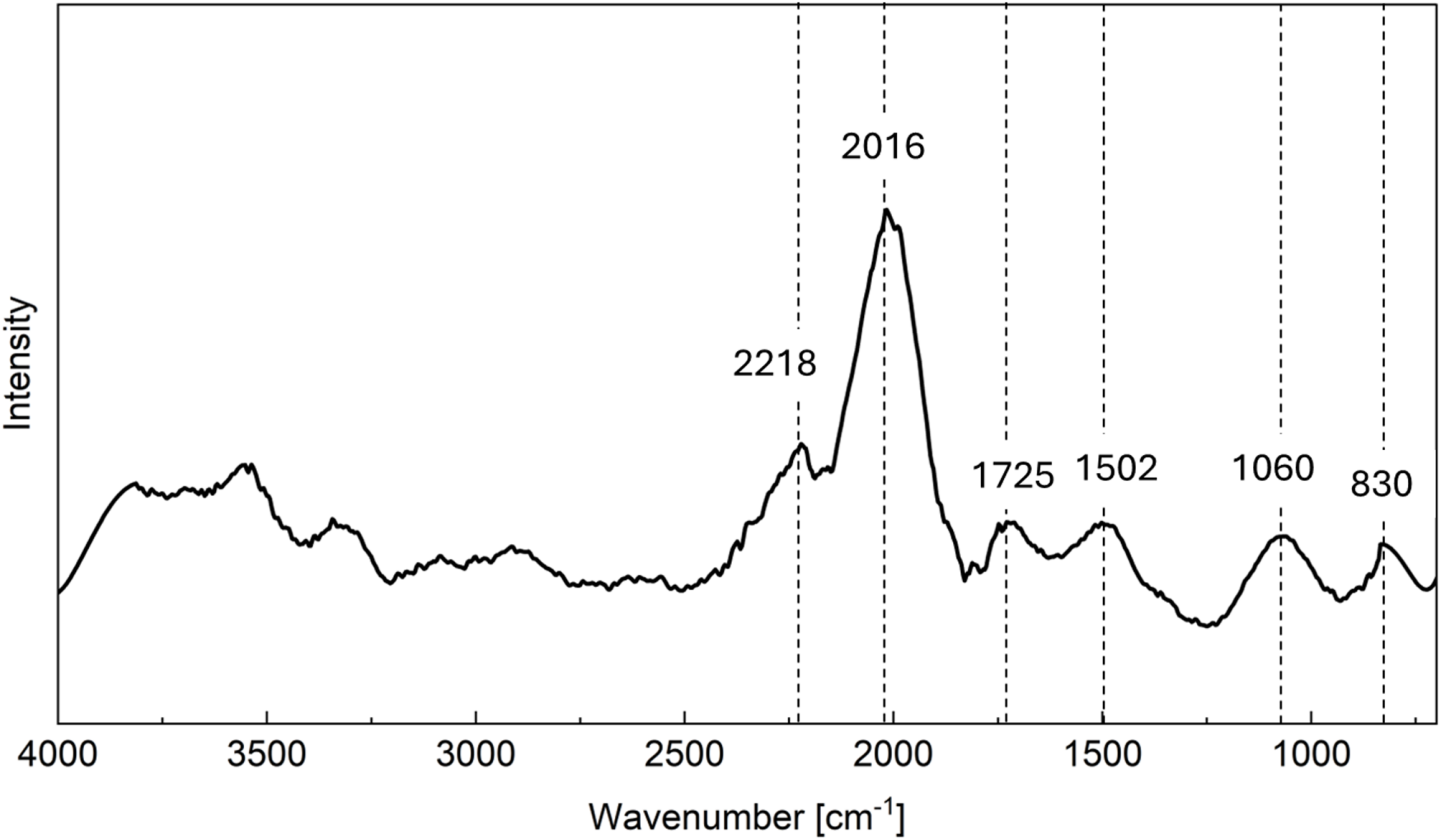
FTIR spectrum of suspended solids collected after centrifugation.

The FTIR spectrum of the suspended solids from centrifugation method was recorded in the range of 400–4000 cm^− 1^ after baseline correction and smoothing (Fig. 4). Several characteristic peaks were identified. The peak at 830 cm^− 1^ is attributed to the Si–O bending vibration, further confirming the presence of silicate or aluminosilicate structures. A band at 1060 cm^− 1^ corresponds to the Si–O–Si asymmetric stretching vibration, which is commonly observed in silicate minerals and silica-rich phases^39^. In addition to these inorganic signals, other absorption bands were observed at 1502, 1725, 2016, and 2218 cm^− 1^. The weak peak observed between at 1725 cm^− 1^ may correspond to C–H bending vibrations in aromatic compounds. The sharp peak at 2016 cm^− 1^ is likely associated with N = C=S stretching vibrations, characteristic of isothiocyanate groups. Additionally, the weak peak at 2218 cm^− 1^ can be attributed to C ≡ C stretching in alkynes or C ≡ N stretching in nitrile functional groups^40^.

**Fig. 5:**
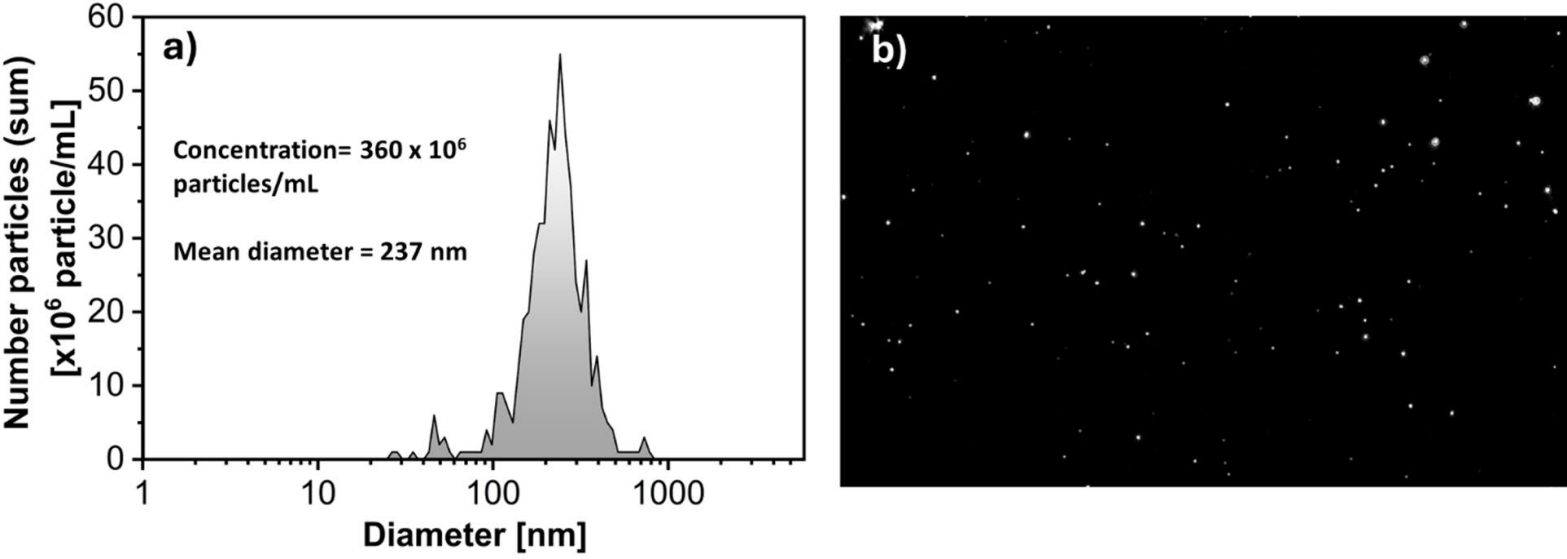
NTA results of mine water diluted 5-fold: (**a**) particle size distribution and (**b**) micrograph of suspended particles.

Nanoparticle tracking analysis (NTA) was performed on the mine water. The results revealed a mean particle diameter of 237 nm, with a particle concentration of 3.6 × 10⁸ particles/mL. The zeta potential was measured at − 29.24 ± 0.9 mV. This moderately stable negative charge is highly diagnostic of the cristobalite and albite surfaces identified via XRD at near-neutral pH^41,42^. These values confirm the presence of a high concentration of sub-micron particles with sufficient surface charge to remain well-dispersed in the solution. Figure 5 shows the results of NTA performed on the mine water diluted five times. The figure presents (a) the particle size distribution and (b) a micrograph of the suspended particles, providing insight into their size range and morphological characteristics.

The combined results of XRF, SEM–EDS, XRD, FTIR, and NTA analyses confirm that the mine water contains naturally occurring mineral particles, cristobalite and albite, that provide stable, negatively charged surfaces (–29.24 mV), which serve as effective sites for heterogeneous nucleation. These findings suggest that naturally occurring particles in mine water can serve as effective nucleation sites, reducing the induction time for hydrate formation and potentially enhancing the overall kinetics of the process. Unlike synthetic or externally added promoters, which often require post-treatment separation and recovery, the suspended solids in mine water are an inherent part of the matrix. Therefore, their use as *in situ* promoters eliminates concerns related to recovery and simplifies the process design for HBD systems.

### Effect of stirring rate on hydrate formation

**Fig. 6:**
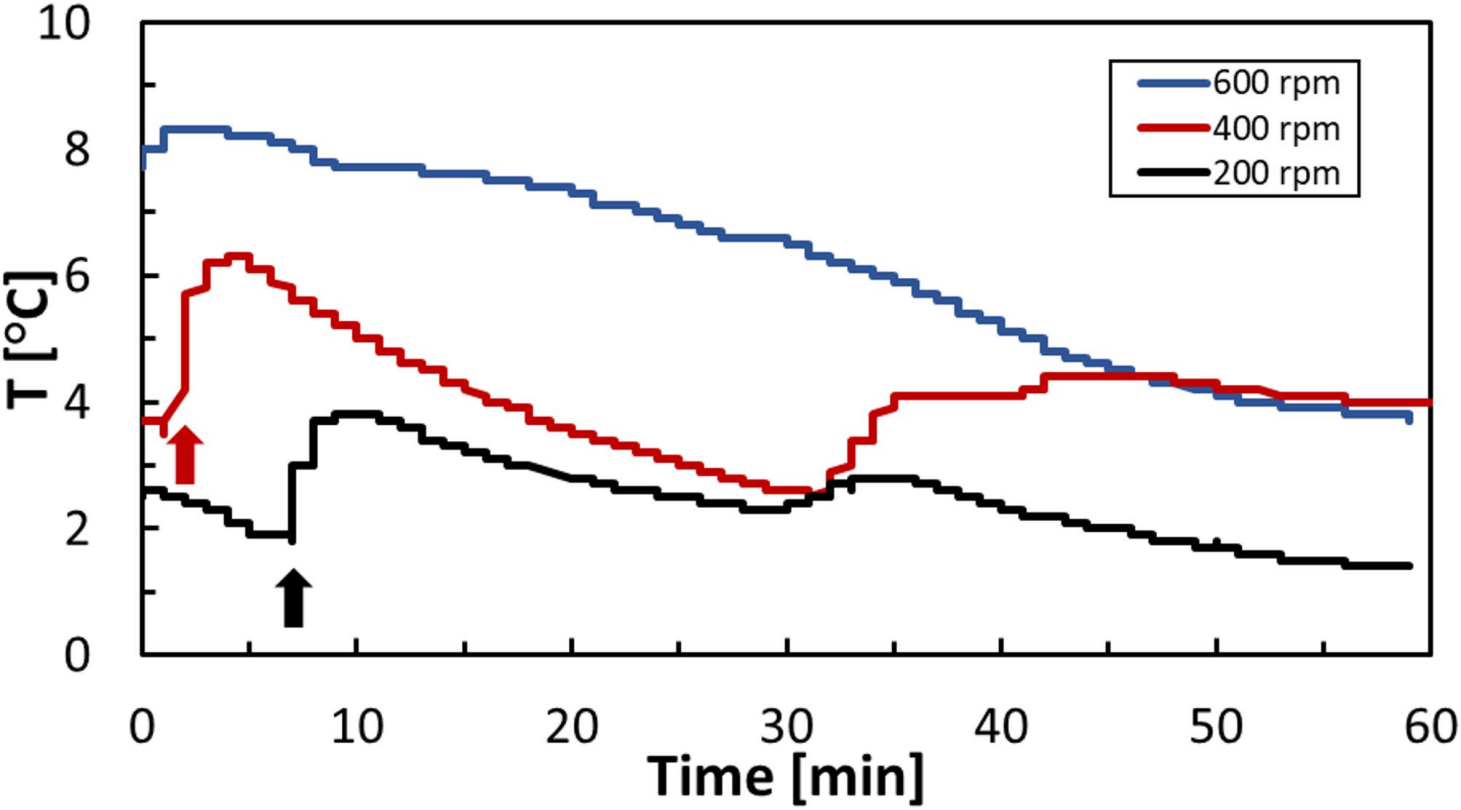
Effect of stirring rate (200, 400, and 600 rpm) on gas hydrate induction time at 274.15 K and 3.58 MPa, for 1 h.

In the series of experiments where only the agitation rate was varied, a higher stirring speed was observed to shorten the induction period. These experiments were conducted using as-received mine water containing naturally occurring suspended solids, at 274.15 K and 3.58 MPa, with a reaction time of 1 h. As shown in Fig. 6, increasing the stirring rate from 200 rpm to 600 rpm reduced the induction time from approximately 8 min to near zero. This behavior is primarily attributed to enhanced turbulence at higher stirring rates, which promotes the dispersion of CO₂ into finer bubbles, increases gas–liquid interfacial area, and accelerates mass transfer, thereby enhancing the kinetics of hydrate nucleation and growth^43–45^. Within the investigated agitation range (200–600 rpm), this kinetic enhancement is the dominant effect. Although extreme agitation has been reported to potentially destabilize pre-nucleation clusters by increasing local disorder^46^, such effects were considered negligible under the operating conditions examined in this study.

**Fig. 7:**
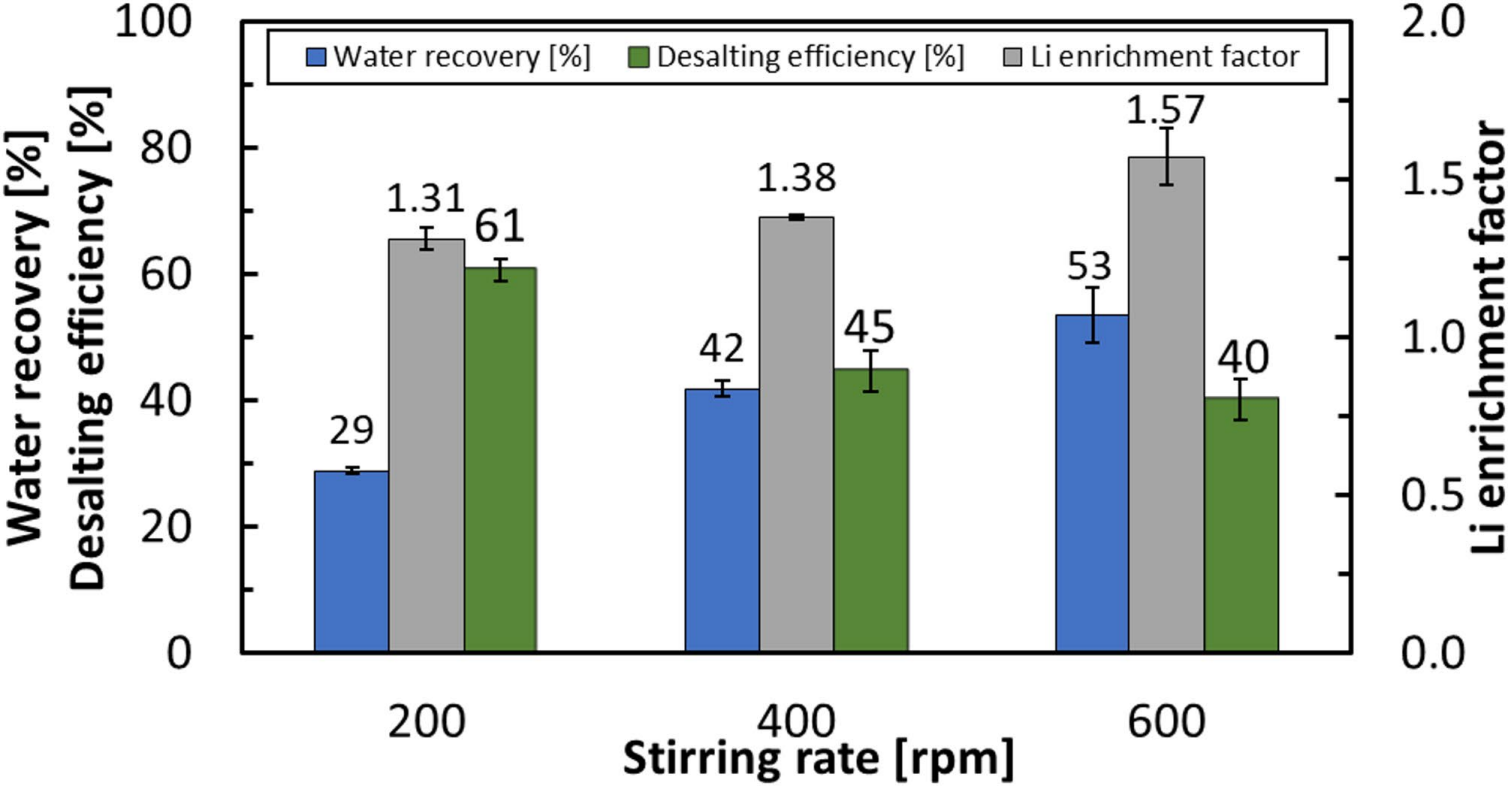
Effect of stirring rate (200, 400, and 600 rpm) on process efficiency under hydrate formation conditions of 274.15 K and 3.58 MPa, with a total duration of 1 h.

In addition to induction time, the impact of stirring rate on key performance indicators was evaluated at 200, 400, and 600 rpm. Increasing the stirring rate from 200 to 600 rpm increased water recovery from 29% to 53% and raised the Li enrichment factor in the brine from 1.31 to 1.57, while the desalting efficiency of the hydrate decreased from 61% to 40% (Fig. 7). Higher agitation enhances gas–liquid mass transfer by dispersing CO_2_ into finer bubbles and increasing the interfacial area, which accelerates hydrate nucleation and growth and thereby increases the rate at which water is incorporated into the hydrate lattice. However, rapid crystal growth reduces the effectiveness of solute exclusion, allowing salts to be trapped within the hydrate phase or adhered to particle surfaces. This reduces hydrate purity and complicates phase separation. Consequently, although a greater fraction of feedwater is converted to hydrate (higher water recovery), the residual brine becomes more concentrated, producing an increased Li enrichment factor despite diminished salt rejection. The aforementioned observed trends are summarized in Fig. 7, confirming the critical role of stirring in HBD process. A stirring rate of 600 rpm was identified as the optimal condition for achieving enhanced kinetic and separation performance and was therefore selected for all subsequent experiments in this work.

### Effect of reaction time on the process’ performance

**Fig. 8:**
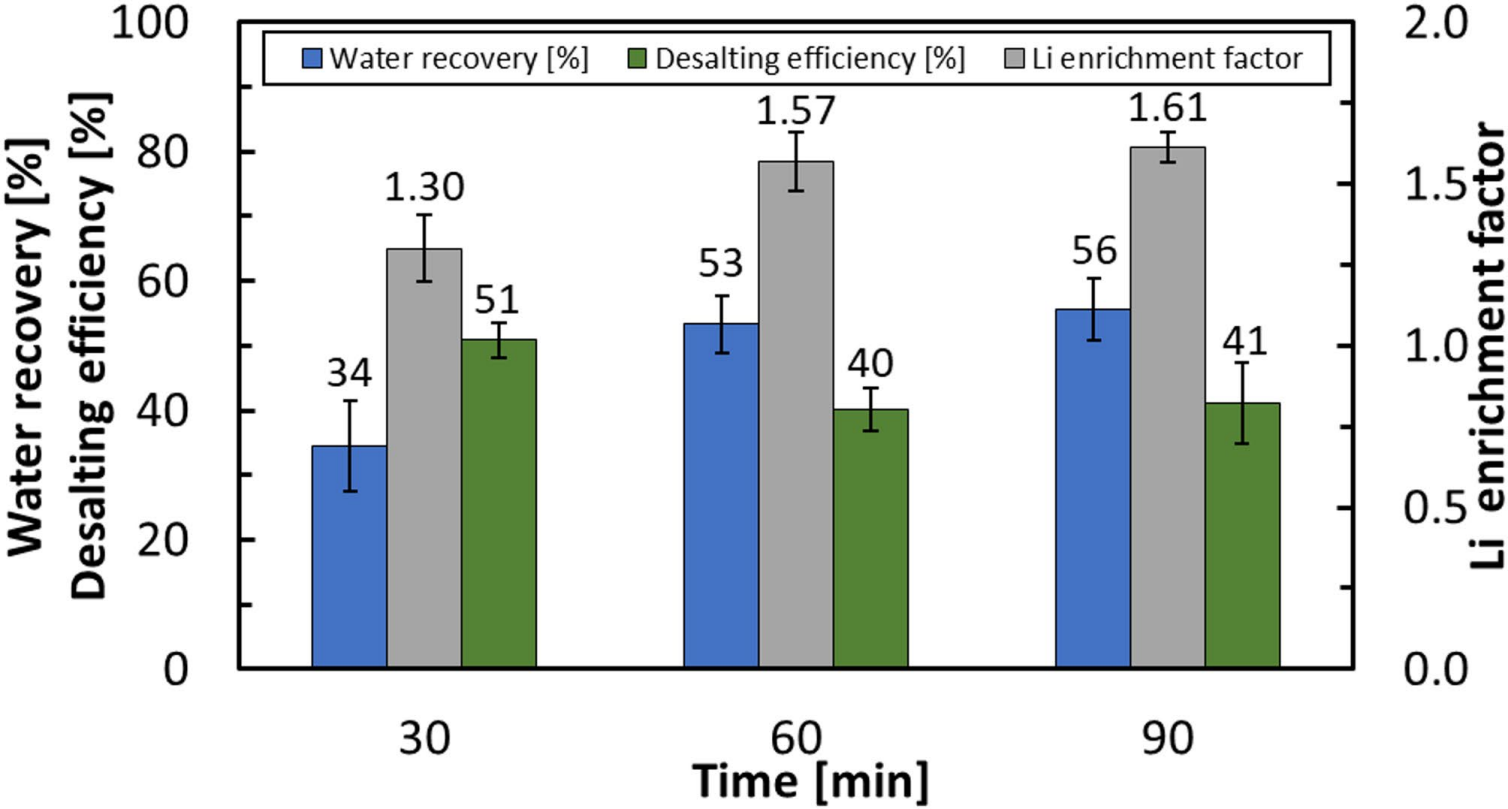
Effect of reaction time (30, 60, and 90 min) on process efficiency under hydrate formation conditions of 274.15 K, 3.58 MPa, and 600 rpm.

The impact of reaction time on HBD performance was investigated at 274.15 K, 3.58 MPa, and a stirring rate of 600 rpm by varying the reaction duration to 30, 60, and 90 min. As shown in Fig. 8, increasing the reaction time from 30 to 60 min improved performance. Water recovery rose from 34% to 53%, while the Li enrichment factor in the residual brine increased from 1.30 to 1.57. In contrast, the desalting efficiency decreased from 51% to 40%, a trend consistent with extended hydrate growth. Longer growth periods increase the probability of salt entrapment within the hydrate lattice, thereby reducing hydrate purity and lowering salt rejection efficiency. Extending the reaction time from 60 to 90 min did not yield further improvements, indicating that the system had reached equilibrium. Moreover, prolonged stirring produced more rigid hydrate structures, making the physical separation of hydrate from brine difficult. Previous reports also suggest that extended residence times can promote partial hydrate decomposition under stirring^47^, which further limits the benefits of longer reaction durations. Accordingly, 60 min was identified as the optimal reaction time, providing a balance between high water recovery and improved Li enrichment, while avoiding the drawbacks of salt entrapment, difficult phase separation, and possible hydrate decomposition at extended durations.

### Multi-stage desalination performance

**Fig. 9:**
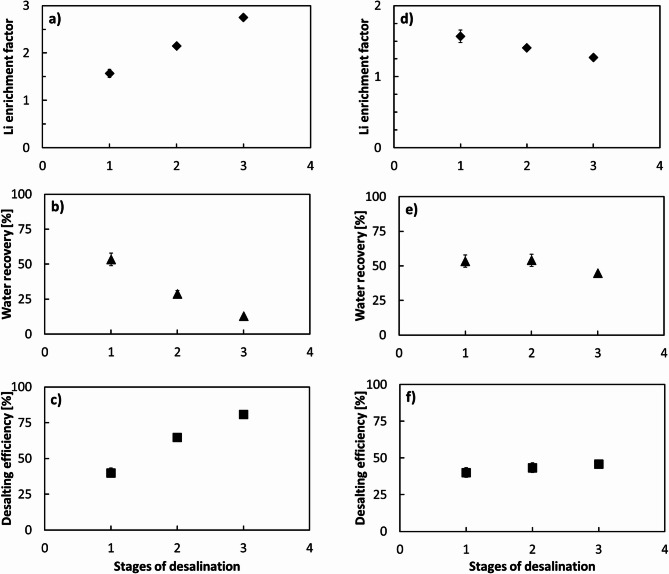
Stage-by-stage and cumulative performance of the multi-stage HBD process conducted as successive batch experiments under hydrate formation conditions of 274.15 K, 3.58 MPa CO_2_ pressure, 600 rpm for 1 h per stage. In each stage, 400 mL of feedwater was treated. Lithium enrichment factor (**a**,**b**), water recovery (**c**,**d**), and desalting efficiency (**e**,**f**) are shown as a function of desalination stage number.

To comprehensively assess the performance of the multi-stage gas HBD process, both individual stage and cumulative results were evaluated for the hydrate and brine streams. The brine stream was collected at each stage, and the Li enrichment factor was calculated by comparing the lithium concentration in the brine to that of the feed for each respective stage. A cumulative Li enrichment factor was also reported by comparing the final brine from stage 3 with the initial mine water, to capture the total lithium concentration increase throughout the process. In parallel, for the hydrate phase, the desalting efficiency was calculated at each stage by comparing the composition of the hydrate melt with the corresponding feed entering that stage. Cumulative values were also determined by comparing the final hydrate (from stage 3) to the initial mine water feed, reflecting the overall purification performance across the integrated process. This dual evaluation approach provides a complete picture of both clean water recovery and Li enrichment potential within the multi-stage system. The stage-by-stage and overall performance of the multi-stage HBD process are presented in Fig. 9.

For the brine stream, Li enrichment factors decreased slightly at each successive stage due to the progressively higher lithium concentration in the feed. Nevertheless, the cumulative enrichment factor increased substantially, reaching 2.75 compared to 1.57 in a single stage operation. In practical terms, this corresponded to an increase in lithium concentration from approximately 180 mg/L in the initial mine water to about 500 mg/L after three stages. This level falls within the concentration range reported in the literature as feasible for further processing and precipitation-based recovery^48–50^, underscoring the potential of multi-stage HBD for lithium resource valorization.

**Fig. 10:**
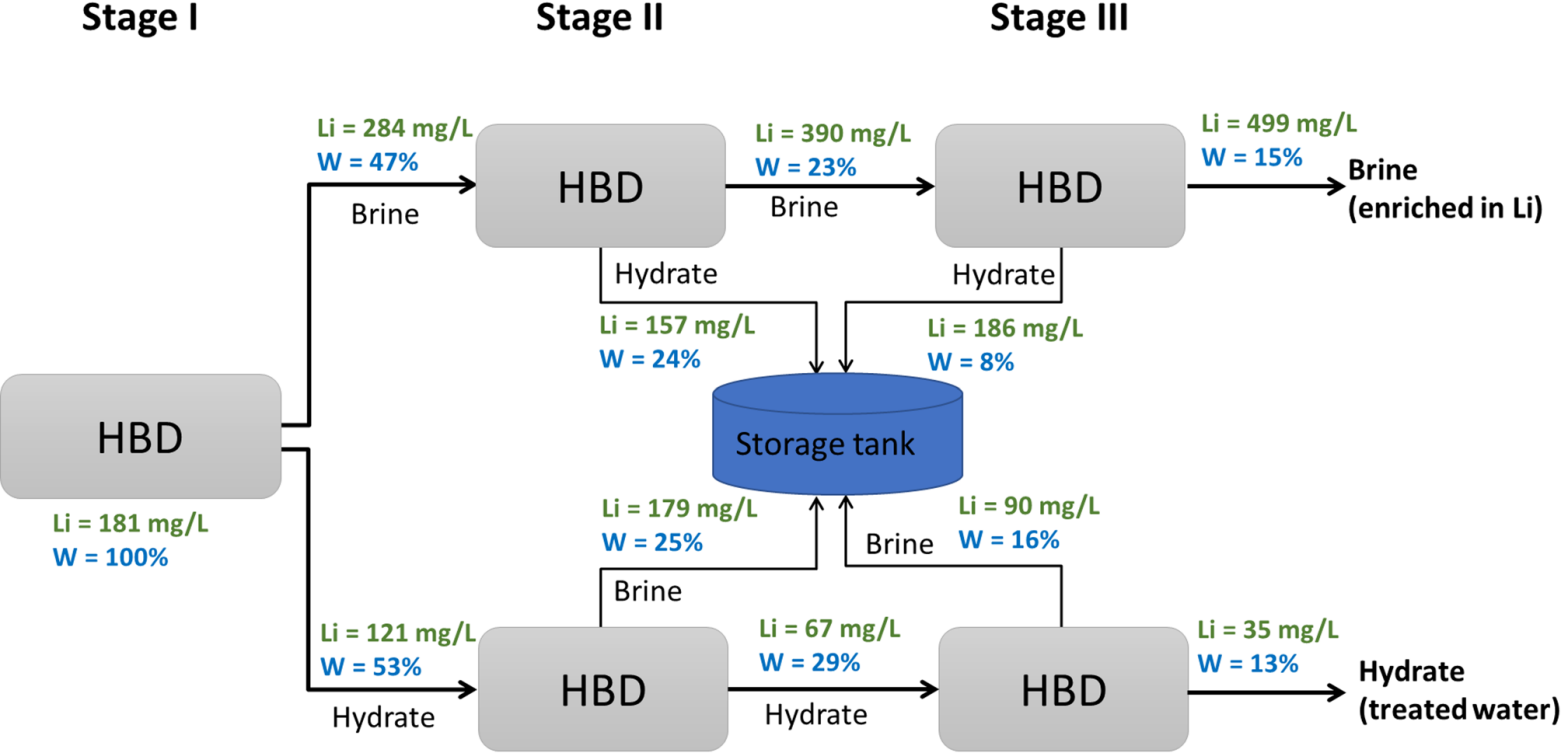
Conceptual illustration of water distribution and lithium concentration evolution across successive stages of the batch multi-stage HBD process. Each stage represents an independent batch experiment conducted under identical operating conditions (400 mL feedwater, 274.15 K, 3.58 MPa CO_2_ pressure, 600 rpm, 1 h reaction time). Arrows indicate stage-to-stage progression of experimental streams and do not represent continuous process flow.

Regarding the hydrate stream, both water recovery and desalting efficiency were analyzed. Water recovery remained close to 50% per stage, but the cumulative recovery across three stages reached 13%. Although the overall water recovery was relatively low, it is worth noting that the concentrated brine collected from stage three of the brine pathway accounted for 15%. This implies that approximately 72% of the original water remained available for recovery in subsequent treatment cycles. The distribution of water fractions and lithium concentrations across the different streams is summarized in Fig. 10. It should be noted that the multi-stage HBD experiments were conducted as successive batch operations, in which hydrate formation was completed and the hydrate phase dissociated at the end of each stage before the resulting streams were used as feedwater for subsequent stages. The lithium concentration in the storage tank was measured at 153 mg/L, suggesting that this fraction could potentially be recycled. However, mixing all residual streams into a single tank would partially dilute the lithium gradient built across stages; therefore, stream-specific recycling strategies would be required in practice to maintain lithium enrichment.

In terms of desalting efficiency, single-stage values were relatively stable at around 40%, but when applied in sequence the cumulative desalting efficiency increased markedly to 81%. As a result, the TDS of the treated water decreased from 3300 mg/L in the initial feed to approximately 640 mg/L in the final hydrate melt. This significant reduction indicates that the treated water could potentially be reused in certain mining operations, thereby reducing freshwater demand. Taken together, the multi-stage HBD process demonstrates dual benefits: progressive concentration of lithium in the brine to levels sufficient for recovery, and substantial improvement in water quality through cumulative desalting.

### Effect of lithium concentration on the process’ performance

The influence of initial lithium concentration on HBD performance was evaluated using mine water at its original lithium level (180 mg/L Li, ≈ 0.03 M) and after LiCl spiking to obtain higher Li concentrations of 0.10 M, 0.23 M, and 0.45 M. At the lowest concentration (0.03 M, as-received), the system achieved the best overall performance, with a Li enrichment factor of 1.57 and water recovery of 53%. As lithium concentration increased to 0.10 M and 0.23 M, both enrichment factor and water recovery declined slightly, while desalting efficiency improved modestly. This increase in desalting efficiency may be linked to the slower hydrate formation kinetics observed at higher salt concentrations. Slower growth can promote the formation of more ordered hydrate structures that trap fewer ions. As a result, hydrates formed under these conditions were slightly purer.

At the highest concentration tested (0.45 M), the adverse effect of salinity became more pronounced. Lithium enrichment decreased to 1.27, and water recovery dropped significantly to 27%. This decline in water recovery can be attributed to the reduced activity of water molecules in highly saline solutions, which makes it more difficult for hydrates to form and requires longer times for crystallization to proceed^15^. The reduction in water recovery and Li enrichment at high salinity is consistent with literature^15^ reports showing that elevated ionic strength suppresses hydrate nucleation and growth by lowering the thermodynamic driving force and limiting the extent of selective ion exclusion.

**Fig. 11:**
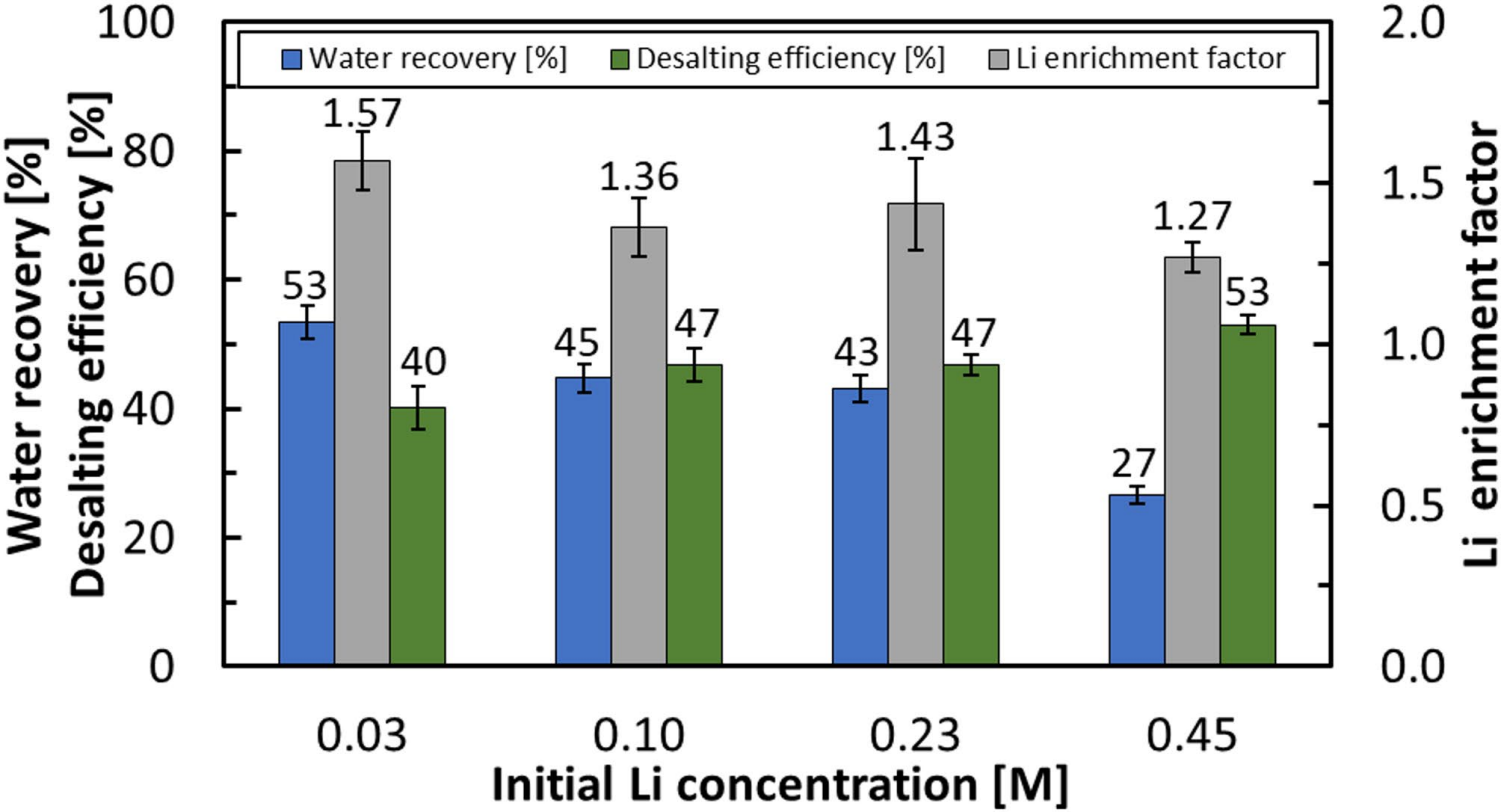
Effect of initial lithium concentration on single-stage process efficiency under hydrate formation conditions of 274.15 K, 3.58 MPa, and 600 rpm for 1 h.

Conversely, desalting efficiency increased from 40% to 53% as the initial Li concentration rose from 0.03 M to 0.45 M. The observed rise in desalting efficiency at higher lithium concentrations may also be explained by salt-induced destabilization effects. In concentrated systems, salts adsorbed at the hydrate surface enhance local disorder and promote partial pre-melting, a phenomenon analogous to the freezing-point depression of ice in saline solutions^51,52^. As a result, hydrate decomposition is favored in highly saline environments, and fewer salts are incorporated into the hydrate lattice, yielding an apparently higher desalting efficiency. Moreover, because fewer hydrates are formed, a larger fraction of the brine remains unconverted. Therefore, the brine becomes less concentrated, leading to a higher apparent desalting efficiency in the hydrate phase. Figure 11 summarizes these trends.

### Comparative performance and practical implications for lithium recovery

To assess the practical relevance of the proposed HBD process, its performance was compared with established lithium concentration technologies, including RO, FO, ED, and NF, as summarized in Table 5. These technologies rely primarily on membrane separation or electro-driven transport mechanisms and have demonstrated high lithium enrichment or recovery under controlled conditions. However, their performance is often constrained by feed salinity, osmotic pressure, scaling, and membrane fouling, particularly in complex industrial or mining effluents.

RO processes can achieve relatively high lithium enrichment factors and water recovery, but increasing feed salinity increases osmotic pressure, reduces permeate flux, and increases specific energy consumption^12^. Similarly, ED offers high lithium recovery and permselectivity in synthetic or pretreated brines, yet its energy consumption increases significantly at high salinities, and long-term operation is limited by membrane scaling and fouling^17^. FO has been explored for lithium concentration with moderate success; although it operates at low hydraulic pressure and exhibits reduced fouling tendencies, it generally suffers from the need for an additional energy-intensive step to regenerate the draw solution^19^. NF shows promising Li/Mg selectivity, but it also requires elevated pressure and careful pretreatment to mitigate membrane degradation^53^.

HBD reported in previous studies has demonstrated lithium enrichment in synthetic systems, often requiring external promoters, such as CO_2_ nanobubbles^16^, graphite^15^, or other additives to reduce induction time and enhance hydrate growth. While effective, these approaches introduce additional process complexity and potential downstream separation requirements. In contrast, the HBD process developed in this work was applied directly to real mine water and achieved rapid hydrate nucleation within 1 h of operation per stage, without the addition of external promoters. Notably, a lithium enrichment factor of 1.57 was obtained after a single 1 h stage, increasing to 2.75 after three successive 1 h stages. While the overall operating time is comparable to the 3 h system reported in^16^, it remains substantially shorter than other systems reported in literature, which often require up to 24 h.

Despite its promising potential for lithium enrichment and water recovery, several challenges must be addressed before CO_2_–HBD can be translated to industrial scale. Mechanical stirring, while essential for enhancing gas–liquid mass transfer and reducing induction time, contributes significantly to energy consumption. Future work should therefore explore optimized agitation strategies, including limiting mixing to the nucleation stage or employing lower-energy reactor configurations. Scale-up also introduces complexities related to heat management, hydrate slurry handling, and hydrate–brine separation, highlighting the need for continuous reactor design and improved separation technologies. Efficient CO_2_ recovery and recycling after hydrate dissociation will be critical for process sustainability and cost reduction. Finally, although naturally occurring mineral particles acted as effective *in situ* promoters in this study, their concentration and mineralogy may vary across mining sites. Future research should therefore investigate the role of different mineral types and concentrations in promoting hydrate formation to establish robust design criteria and ensure process reproducibility under variable field conditions.

**Table 5:** Performance and energy comparison of conventional membrane processes and hydrate-based desalination for lithium concentration from saline waters.

Process	Specific feed type	Performance/energy consumption	Key advantages/limitations	Ref.
Reverse osmosis (operating *P* = 3.5 MPa)	Lithium-enriched brine from salt lake brine (Li = 54 mg/L) Other major components: Na = 104 mg/L, BO_3_ =5.1 mg/L	At 80% recovery: Li enrichment = 5.53 Flux = 70.77 L/m^2^.h	Advantages: high water recovery and effective lithium enrichment. Limitations: elevated salinity increases osmotic pressure, concentration polarization, and membrane fouling, leading to reduced permeate flux, lower concentration efficiency, and increased energy consumption.	^12^
Reverse osmosis (operating *P* = 2 MPa)	Industrial lithium-containing wastewater (Li = 1269 mg/L) Other major component: Na = 110 mg/L	After 50 min: Li enrichment = 3.5 Energy consumption = 7.81 kW.h/m^3^		^17^
Forward osmosis	Feed solution: 3,000 mg/L of Li Draw solution: simulated brine solution from Uyuni Salt Lake (Li = 1,414 mg/L) Other major components: Na = 61,358 mg/L, B = 855 mg/L, K = 25,120 mg/L, Mg = 18,600 mg/L	After 30 h: Water flux decreased from 4.7 to 0.4 L/m2.h Li in feed increased from 3,000 to 12,700 mg/L Li in draw decreased from 1,414 to 850 mg/L	Advantages: attractive due to its low fouling potential, operational simplicity, and ability to achieve high water recovery without high-pressure pumping. Limitations: typically characterized by lower water flux (resulting in longer processing times) and the need for an energy-intensive secondary step to regenerate or recover the draw solution.	^13^
Forward osmosis	Feed solution: synthesized feed solution (Li = 2,500 mg/L) Draw solution: deep eutectic solvent (choline chloride: 2 ethylene glycol)	After 6 h: Li in feed increased from 2,500 to 9,200 mg/L		^19^
Electrodialysis (voltage drop = 8 V)	Industrial lithium-containing wastewater (Li = 1,269 mg/L) Other major component: Na = 110 mg/L	After 55 min: Conductivity of concentrated solution increased from 17.87 to 55.20 mS/cm Average flux of Li = 2.73 mol/m^2^.h Energy consumption = 26.67 kW.h/m^3^	Advantages: increasing the applied voltage enhances the driving force for ion migration, significantly reducing processing time and improving lithium transport. Limitations: higher voltage results in increased current density and elevated specific energy consumption. In high-salinity matrices, membrane scaling and fouling are also intensified, leading to increased operational costs and maintenance requirements.	^17^
Electrodialysis (current density = 5.9 A/m^2^)	Synthetic salt lake brine (Li = 150 mg/L) Other major component: Mg = 10,000–60,000 mg/L	After 3 h: Li recovery = 94.5% Permselectivity index S_Li/Mg_ = 20-33 Energy consumption = 1.9 kW.h/Kg Li		^9^
Nanofiltration (operating *P* = 0.8 MPa, NF90 membrane)	Synthetic salt lake brine (Li = 141 mg/L) Other major component: Mg = 1,410 mg/L	After 8 h: Li rejection = 77% Mg/Li molar ratio decreased from 10 to 0.19	Advantages: effective Li/Mg separation due to size and charge-based exclusion mechanisms. Limitations: performance declines at high salinity due to concentration polarization, scaling, and membrane fouling.	^53^
Hydrate-based desalination (hydrate former = CO_2_, T = 274.15 K, *P* = 3.58 MPa, promoter = CO_2_ nanobubbles)	Synthetic lithium solution (Li = 694 mg/L)	After 3 h: Water recovery = 72% Li enrichment = 2.25 Desalination efficiency = 50%	Advantages: operates without membranes or high hydraulic pressure; capable of simultaneous water recovery and lithium concentration under mild thermodynamic conditions. Limitations: characterized by slow hydrate formation kinetics, the need for promoters to accelerate the process, and challenges in hydrate–brine separation and scale-up.	^16^
Hydrate-based desalination (hydrate former = cyclopentane, T = 275.15 K, *P* = 0.1 MPa, promoter = graphite)	Synthetic lithium solution (Li = 694 mg/L)	After 24 h: Induction time ~ 35 min Water recovery = 72% Li enrichment ~ 1.5 Desalination efficiency = 50%		^15^
Hydrate-based desalination (hydrate former = CO_2_, T = 274.15 K, *P* = 3.58 MPa)	Real mine water (Li = 181 mg/L) Other major components: Na = 937 mg/L, K = 145 mg/L, Mg = 14, Ca = 57 mg/L, SO4 = 2,592 mg/L, Cl = 358 mg/L, carbonated species = 223 mg/L)	After 1 h: Induction Time < 8 min (via *in-situ* promoter) Water recovery = 53% Li enrichment of single stage = 1.57 Li enrichment of three stage = 2.75		This study

## Conclusions

This study demonstrated the feasibility of CO_2_ HBD for simultaneous Li enrichment and recovery of clean water from real mine waters under mild conditions (274 K, 3.58 MPa). A key finding is that naturally occurring suspended solids (silicate/aluminosilicate particles) present in the mine water, even at low concentrations (15 mg/L), function as effective *in situ* kinetic promoters, accelerating hydrate nucleation and thereby eliminating the need for external additives. This external promoter-free mechanism simplifies the process and reduces potential downstream separation and operational costs.

Stirring was shown to be a critical operational parameter. Increasing the rate from 200 to 600 rpm reduced induction time from 8 min to nearly zero, while improving water recovery from 29 ± 1% to 53 ± 4% and enhancing Li enrichment in the brine from 1.31 ± 0.04 to 1.57 ± 0.09. Reaction time optimization revealed that 60 min provided the best balance of recovery and enrichment, with longer durations yielding no further gains and even complicating hydrate–brine separation. Similarly, initial lithium concentration influenced performance: the as-received mine water (0.03 M Li) gave the highest Li enrichment and water recovery (1.57 ± 0.09 and 53 ± 4%, respectively), while higher lithium concentration (0.45 M) suppressed hydrate formation, lowering water recovery to 27 ± 3% and reducing Li enrichment factor to 1.27 ± 0.05.

A multi-stage configuration further highlighted the dual benefits of HBD. For the brine stream, Li enrichment reached 2.75 after three stages, increasing concentration from 180 to 500 mg/L, within the practical range for precipitation-based recovery. For the hydrate stream, cumulative desalting efficiency rose from 40% in a single stage to 81% after three stages, with TDS reduced from 3300 to 640 mg/L, suggesting reuse potential in mining operations. Taken together, these findings establish CO_2_ hydrate-based desalination as a promising approach for sustainable mine water management, providing both resource recovery and water reuse without reliance on chemical promoters.

## Data Availability

All data generated or analyzed during this study are included in this article.

## Abbreviations

ATR: Attenuated total reflectance
ED: Electrodialysis
EDS: Energy dispersive spectroscopy
FO: Forward osmosis
FTIR: Fourier-transform infrared spectroscopy
HBD: Hydrate-based desalination
HPIC: High-pressure ion chromatography
ICP-OES: Inductively coupled plasma optical emission spectrometry
MP-AES: Microwave plasma atomic emission spectroscopy
NF: Nanofiltration
NTA: Nanoparticle tracking analysis
RO: Reverse osmosis
SEM: Scanning electron microscopy
SS: Suspended solids
TDS: Total dissolved solids
TOC: Total organic carbon
XRF: X-ray fluorescence
